# The progestational and androgenic properties of medroxyprogesterone acetate: gene regulatory overlap with dihydrotestosterone in breast cancer cells

**DOI:** 10.1186/bcr1340

**Published:** 2005-11-02

**Authors:** Radhika P Ghatge, Britta M Jacobsen, Stephanie A Schittone, Kathryn B Horwitz

**Affiliations:** 1 University of Colorado Health Sciences Center, Department of Medicine, Division of Endocrinology, Metabolism and Diabetes, Denver, Colorado, USA

## Abstract

**Introduction:**

Medroxyprogesterone acetate (MPA), the major progestin used for oral contraception and hormone replacement therapy, has been implicated in increased breast cancer risk. Is this risk due to its progestational or androgenic properties? To address this, we assessed the transcriptional effects of MPA as compared with those of progesterone and dihydrotestosterone (DHT) in human breast cancer cells.

**Method:**

A new progesterone receptor-negative, androgen receptor-positive human breast cancer cell line, designated Y-AR, was engineered and characterized. Transcription assays using a synthetic promoter/reporter construct, as well as endogenous gene expression profiling comparing progesterone, MPA and DHT, were performed in cells either lacking or containing progesterone receptor and/or androgen receptor.

**Results:**

In progesterone receptor-positive cells, MPA was found to be an effective progestin through both progesterone receptor isoforms in transient transcription assays. Interestingly, DHT signaled through progesterone receptor type B. Expression profiling of endogenous progesterone receptor-regulated genes comparing progesterone and MPA suggested that although MPA may be a somewhat more potent progestin than progesterone, it is qualitatively similar to progesterone. To address effects of MPA through androgen receptor, expression profiling was performed comparing progesterone, MPA and DHT using Y-AR cells. These studies showed extensive gene regulatory overlap between DHT and MPA through androgen receptor and none with progesterone. Interestingly, there was no difference between pharmacological MPA and physiological MPA, suggesting that high-dose therapeutic MPA may be superfluous.

**Conclusion:**

Our comparison of the gene regulatory profiles of MPA and progesterone suggests that, for physiologic hormone replacement therapy, the actions of MPA do not mimic those of endogenous progesterone alone. Clinically, the complex pharmacology of MPA not only influences its side-effect profile; but it is also possible that the increased breast cancer risk and/or the therapeutic efficacy of MPA in cancer treatment is in part mediated by androgen receptor.

## Introduction

Synthetic progestins have been in use since the 1950s. Today more than 15 million women use them either in combination oral contraceptives or for hormone replacement therapy (HRT) [1]. Although progestins are added to estrogens because they significantly lower the risk for endometrial cancer, HRT is often not started or discontinued prematurely because of breast cancer fears. Recent data from the Women's Health Initiative [2,3] suggest slight increases in relative risk (RR) for developing invasive breast cancer (RR 1.26), cardiovascular disease (RR 1.22), pulmonary emboli (RR 2.13), and dementia (RR 2.05) in women on HRT containing continuous dosing of conjugated equine estrogen and medroxyprogesterone acetate (MPA). This led to premature termination of the estrogen + MPA arm of the trial. The estrogen-only arm was recently terminated and its analysis, although indicating an increased risk for stroke (hazard ratio 1.39), showed no increase in breast cancer risk (hazard ratio 0.77) [4]. These and other data [5] implicate the progestin component in the increased breast cancer risk observed with estrogen + MPA [6].

**Figure 1:**
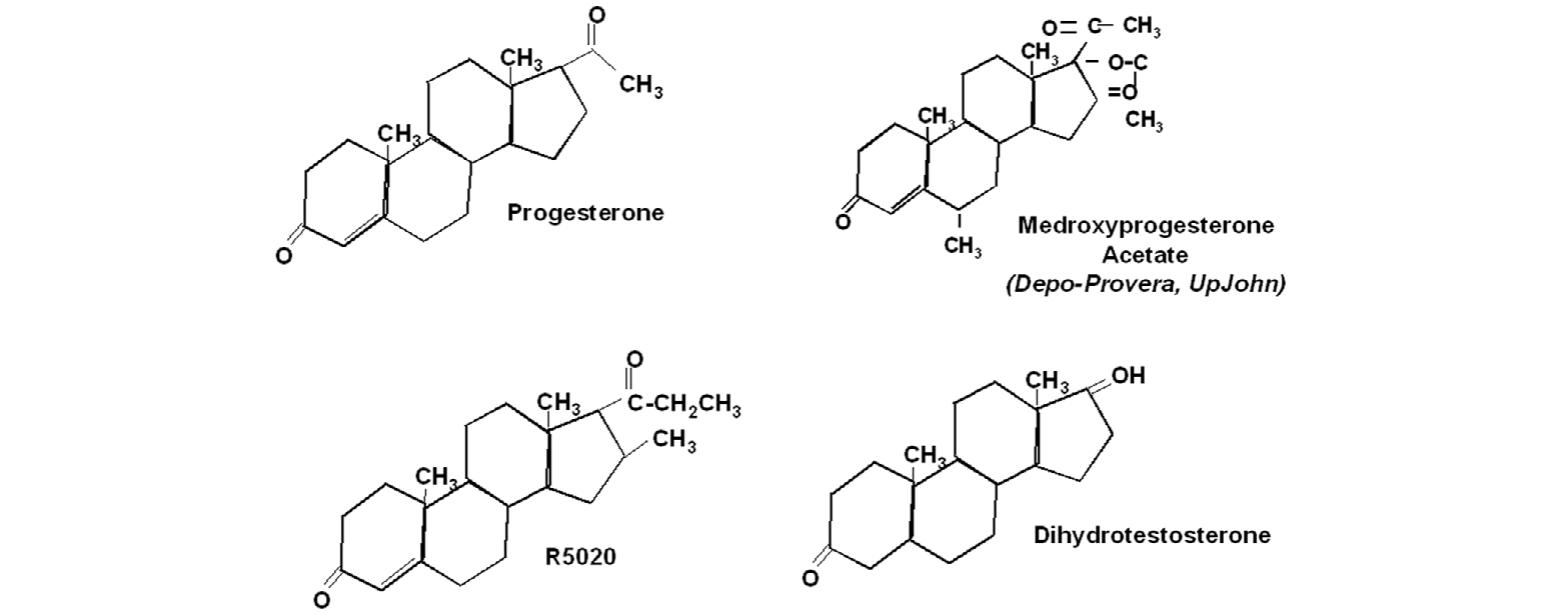
Chemical structures of progesterone, MPA, R5020 and DHT. The pharmaceutical name and manufacturer are shown italic text. DHT, dihydrotestosterone; MPA, medroxyprogesterone acetate.

Most synthetic progestins are derived from either testosterone or 17α-hydroxyprogesterone (Fig. 1). MPA (Depo-Provera UpJohn, St Louis, MO and Provera UpJohn, St Louis, MO) is a 17α-hydroxyprogesterone derivative and is the most common HRT progestin in use in the USA [7]. It is used in place of progesterone because of its longer half-life [7]. Synthetic progestins in general were originally selected based on the similarity of their pharmacology to that of progesterone [7]. However, although modern molecular information is available about the genes regulated by progesterone [8,9], there are no global data on the genes regulated by synthetic progestins. Recently, Nilsen and Brinton [10] reported differences between the abilities of MPA and progesterone to activate mitogen-activated protein kinase signaling in hippocampal neurons. Progesterone and MPA also differ in their ability to express vascular cell adhesion molecule-1 in human vascular endothelial cells [11]. Furthermore, in addition to their progestational effects, and unlike natural progesterone, synthetic progestins often bind other steroid receptors including those for androgens (androgen receptors [AR]) and glucocorticoids (glucocorticoid receptors [GR]) [12,13]. Thus, depending on the dose and tissue, it is possible that synthetic progestins differ in their gene regulatory profile compared with progesterone.

Progestins signal mainly though progesterone receptors (PR), which are members of the steroid receptor family of nuclear receptors. There are two naturally occurring human PR isoforms, PR-A and PR-B [14,15], which are identical except for 164 additional amino acids at the amino-terminus of PR-B. For this reason, the two PR vary in their ability to activate transcription on exogenous [16-20] and endogenous promoters, with PR-B being 10 times more active than PR-A [8,9,19]. Ratios of PR-A to PR-B ratios vary in different tissues, physiological states, and breast cancers [21-24]. The normal human breast contains equimolar amounts of PR-A and PR-B, but ratios of PR-A to PR-B are dysregulated in more than 70% of advanced breast cancers [25]. Excess PR-A levels correlate with poorer disease-free survival and more rapid relapse following tamoxifen treatment [26]. Ligand binding leads to PR activation, dimerization, and binding of dimers to promoters at progesterone responsive elements (PREs), followed by interaction with coregulators and altered transcription [27]. PREs are also consensus binding sites for other nuclear receptors, including AR and GR [28-30]. In additional, given the 60% similarity in the ligand binding domains between AR and PR, some ligands – including MPA – can bind both steroid receptors [31].

*In vivo*, progesterone and most natural steroid hormones are actively metabolized in the liver [32]. In breast cancer cells *in vitro*, progesterone at physiologic concentrations (10–20 nmol/l) is sufficient to activate all receptors [33]. Physiologic concentrations of progesterone have a half-life of 2–4 hours in cells, and even at pharmacologic concentrations (1 μmol/l) progesterone is still entirely metabolized within 18 hours to its final metabolic product, namely 5α-pregnan-3β, 6α-diol-20-one it's correct[34]. In contrast, under the same *in vitro *conditions synthetic progestins, including MPA, megestrol acetate, R5020, and the antiprogestin RU486, are not metabolized at all [35], and 20–40% of dihydrotestosterone (DHT) remains unmetabolized at 18 hours [34]. Specifically with regard to MPA, the active form is the parent compound MPA itself [7]. Serum levels of MPA needed for contraceptive efficacy are about 2 nmol/l (1 ng/ml) [36] and for HRT the range is 0.02–0.2 nmol/l (0.01–0.1 ng/ml); these levels are considered to be 'physiologic'. In addition to its use in contraception and HRT, MPA is also commonly used to treat endometrial cancer and as a second line agent to treat breast cancer [37]; for these indications its serum levels are approximately 0.14–1.7 μmol/l (55–650 ng/ml) [38,39], which are 'pharmacologic' concentrations. Thus, depending on need, there is a wide range of concentrations at which MPA is used clinically. MPA also has known glucocorticoid [40,41] and androgenic [40,42,43] activities in breast cancer cells. It exhibits moderate relative binding affinity (58%) to GR and a slightly slower relative binding affinity to AR (36%) [44].

The goal of the present study was to evaluate the transcriptional similarities and differences between physiologic and pharmacologic progesterone and MPA concentrations, focusing on the abilities of these hormones to signal through PR, AR, and GR. Under the conditions tested, we found that – in addition to its progestational actions – MPA is an active androgen. We therefore defined global gene regulatory patterns of MPA on PR and AR and showed that at physiologic concentrations and early time points MPA is a dual function hormone. Like progesterone, it is a good progestin; unlike progesterone, it is also a strong androgen in breast cancer cells. These findings may have important clinical implications for physicians counseling women regarding the use of MPA in HRT.

## Materials and methods

### Cell lines and culture

The T47Dco breast cancer cell line (PR-A^+ ^and PR-B^+^), its PR-negative T47D-Y subline, and construction of T47D-YB (PR-B^+^) and T47D-YA (PR-A^+^) cell lines were previously described [45,46]. Construction of HeLa cervical carcinoma cells stably expressing flag tagged PR-A (HeLa-A) or PR-B (HeLa-B) were also previously described [47]. To construct Y-AR cells, PR-negative T47D-Y cells were electroporated with a G418 resistance plasmid along with the pCMV5-AR vector containing the complete sequence for human AR [48] (a kind gift from E Wilson). Colonies were picked and maintained under G418 selection. Transient transfection with a PRE_2_/luciferase reporter [49] and western blotting for AR were used to screen 19 clones, using T47D-Y cells transiently transfected with both AR and the reporter as a positive control. For this, cells were treated with ethanol or 1 μmol/l DHT and harvested at 20 hours, and relative luciferase activity was assessed. Three positive clones were identified, and clone 17 (renamed Y-AR) was used for further experiments. Cells were routinely passaged in minimum essential medium with Earle's salts containing L-glutamine (292 μg/l) buffered with sodium bicarbonate (2.2 μg/l), insulin (6 ng/ml), and 7.5% fetal calf serum. Cells stably expressing receptors – YB, Y-AR, HeLa-A and HeLa-B – were also cultured in 200 μg/ml G418.

### Transcription assays

Progesterone and MPA were obtained from Sigma (St Louis, MO, USA) and DHT was a gift from M Wierman (Veterans Affairs Medical Center, Denver, CO, USA). Cells were placed in minimum essential medium containing 5% twice dextran-coated charcoal (DCC) stripped serum for 24 hours. They were then transfected by electroporation at 220 V and 950 μF with the PRE_2_/TATA-luciferase reporter along with CMV/Renilla as an internal control, and with PR-B, PR-A, or AR expression vectors as appropriate. Cells were plated at a density of 10^6 ^cells/35 mm dish and treated with increasing concentrations of hormones in triplicate. The cells were harvested 20 hours later, lysed in 1× lysis buffer (Promega, San Diego, CA, USA) and luciferase and Renilla activity (Promega, Madison, WI, USA) were measured. Prism v3.0 (GraphPad Software, San Diego, CA, USA) was used for all data analysis. Statistical analysis was performed using the Student's t-test. Error bars reflect the standard error of the mean. HeLa wild-type, HeLa-A, and HeLa-B cells were plated in 60 mm dishes at 120,000 cells/dish in 3 ml of 5% twice DCC-stripped serum containing medium. HeLa cells stably expressing PR-A or PR-B were transfected with the PRE_2_/TATA-luciferase (1 μg/dish) and SV40/Renilla (10 ng/dish) constructs using CaPO_4_. After overnight incubation at 37°C, precipitate was washed out and cells were treated with 0.01 nmol/l to 1 μmol/l R5020, MPA, or DHT dissolved in ethanol. Cells were harvested at 20 hours, lysed in 1× lysis buffer, and luciferase and Renilla activity measured. Wild-type HeLa cells were also transfected with an AR expression vector (50 ng/dish) as appropriate.

### Expression profiling

T47Dco, Y, and Y-AR cells in log phase were placed in 5% twice DCC-stripped serum containing medium for 24 hours and then treated with vehicle, or 10 nmol/l progesterone or MPA for 6 hours. Y-AR cells were additionally treated with 1 μmol/l MPA and 10 nmol/l DHT. Experiments were done in triplicate for each cell line and treatment group using time separated samples. AR and/or PR protein levels were monitored by immunoblotting. Cells were harvested and washed, and total RNA was isolated using Rneasy Midi Kit (Qiagen, Valencia, CA, USA). RNA integrity was confirmed using an Agilent Bioanalyzer (Agilent, Palo Alto, CA, USA). RNA was prepared according to the Affymetrix Expression Analysis Technical Manual (Affymetrix, Inc., Santa Clara, CA, USA). Briefly, first and second strand cDNA synthesis was performed followed by clean up of double-stranded cDNA. Antisense cRNA was biotin labeled for 4 hours in an *in vitro *transcription reaction, and 20 μg biotin-labeled cRNA was fragmented and hybridized. Microarray analysis was performed using Affymetrix gene chips (HuFL-U133 plus2). Data were analyzed using Gene Spring version 6.0 (Silicon Genetics, San Carlos, CA, USA) and GCOS (Affymetrix, Inc.). Each treatment group was compared with ethanol treatment. Low-dose and high-dose MPA treatments were additionally compared with each other. Statistical significance (*P *< 0.05) for a 2.0-fold change was assessed by one-way analysis of variance. Dendrograms were generated in Gene Spring version 6.0 (Silicon Genetics) using hierarchical clustering, and similarity was measured using a distance correlation [50].

### Androgen receptor immunoblotting

Whole cell extracts were prepared in radioimmune precipitation buffer with protease inhibitors, as described previously [47]. Extracts were resolved on a 7.5% denaturing polyacrylamide gel, transferred to nitrocellulose, and blocked and probed for AR with a 1:3000 dilution of AR antibody (PG-21; Upstate Biotechnology, Lake Placid, NY, USA). Following incubation with a horseradish peroxidase-conjugated goat anti-rabbit secondary antibody, protein bands were visualized by enhanced chemiluminescence (Amersham Biosciences Pharmacia Biotech, Arlington Heights, IL).

### Immunocytochemistry/confocal microscopy

Sterile coverslips were placed into 35 mm dishes along with Y-AR cells at a density of 60,000 cells/35 mm dish in 5% twice DCC-stripped serum overnight. Cells were treated with ethanol or 1 μmol/l DHT for 30 min, 2 hours and 20 hours, and fixed in a 70% methanol/30% acetone mixture for 5 min. Coverslips were rinsed in phosphate-buffered saline (PBS), blocked in 10% normal goat serum/PBS for 1 hour at room temperature, and washed twice in PBS. Coverslips were probed with PG-21 diluted to 0.5 μg/ml in 1% normal goat serum/PBS for 2 hours at 25°C. Cells were washed twice with PBS for 5 min and incubated with a 1:1500 dilution of goat anti-rabbit IgG flourescein-conjugated secondary antibody for 2 hours and then washed. The primary antibody was omitted to control for nonspecific staining. Cells were incubated with 1 ml of a 1:1000 dilution of DAPI in methanol for 15 min at 37°C for nuclear staining. Coverslips were mounted with 50 μl Fluoromount G (Electron Microscopy Sciences, Hatfield, PA, USA). Cells were visualized using an Olympus IX70 inverted microscope and a Photometrics Quantix camera at 100× magnification. Images were deconvolved using a Silicon Graphics O2 computer with DeltaVision software. Representative z stack images are shown.

### Reverse transcription polymerase chain reaction

Regulation of several genes was confirmed by RT-PCR. Y and Y-AR cells were placed in 5% twice DCC-stripped serum overnight, treated with appropriate doses of EtOH, R5020, progesterone, MPA and DHT for 6 or 12 hours, and harvested for RNA (Rneasy Midi Kit, Qiagen). cDNA was synthesized from independent total RNA [8]. Samples were resolved on 2% agarose gels and stained with ethidium bromide. GAPDH (glyceraldehyde-3-phosphate dehydrogenase) was run as an internal control [9]. Primer sets are as described in Table 1.

## Results

### Synthetic progestins and progesterone receptor

Historically, synthetic progestins were developed based on the similarity of their biologic actions to those of progesterone [7]. Figure 1 shows the chemical structure of progesterone and those of its steroidal 17α-hydroxyprogesterone derivatives MPA and R5020, which are used clinically and experimentally, respectively. Also shown is the structure of the androgen DHT [7].

**Figure 2:**
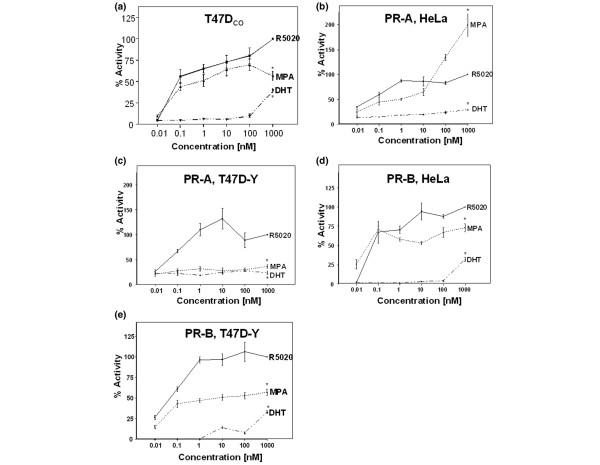
Transcriptional activities of MPA, R5020 and DHT under the control of PR and PR isoforms. **(a) **T47Dco breast cancer cells with wild-type equimolar levels of PR-A and PR-B. T47Dco cells were transfected with PRE_2_/luciferase and a Renilla luciferase internal control. Cells were treated for 20 hours with MPA, R5020, or DHT, at concentrations ranging from 0.01 to 1000 nmol/l. Luciferase activity was quantitated as a percentage of 1 μmol/l R5020 activity. The study was performed in triplicate and data are reported as mean ± standard error. *P < 0.05, 1 μmol/l dose of hormone versus 1 μmol/l R5020. **(b) **HeLa cervicocarcinoma cells stably expressing PR-A. **(c) **PR-negative T47D-Y breast cancer cells transiently transfected with PR-A. **(d) **HeLa cells stably expressing PR-B. **(e) **T47D-Y cells stably expressing PR-B. Transient transfection and transcription studies were performed as described above for panel a. DHT, dihydrotestosterone; MPA, medroxyprogesterone acetate; PR, progesterone receptor.

The transcriptional activity of metabolizable progesterone, when given every 6 hours, is identical to that of 24 hours of nonmetabolizable R5020 (not shown). We therefore initially used 24 hours of R5020 as the standard against which to compare 24 hours of MPA for *in vitro *transcription assays. Wild-type T47Dco breast cancer cells endogenously expressing PR-A and PR-B were transfected with a PRE_2_/luciferase construct and treated with increasing concentrations of R5020, MPA, or DHT. Figure 2a shows approximately equivalent transcriptional activities by R5020 and MPA at all except the 1000 nmol/l dose. DHT had little activity on PR except at the 1000 nmol/l dose.

Although wild-type HeLa cervical cancer cells are both PR and AR negative, T47D-Y (PR-negative) cells did express low levels of endogenous AR by western blot. However, the AR were not functional, based on transcription assays with MPA or DHT, and by RT-PCR transcript expression of the androgen-responsive prostate-specific antigen (PSA) gene (not shown). We next addressed the contribution of each PR isoform individually in HeLa cervical and T47D-Y breast cancer cells exogenously expressing PR-A or PR-B, cotransfected with PRE_2_/luciferase, and treated with increasing concentrations of R5020, MPA, or DHT (Fig 2b–e). Because of differing transfection efficiencies of Y cells transiently transfected with PR-A and YB cells, a comparison of luciferase activities could only be made between hormone treatments in the same cell line. A mock transfected control vector exhibited no activity upon addition of vehicle or hormone (not shown). On PR-A in HeLa cells, MPA had equivalent activity to R5020 at physiologic doses but higher activity at pharmacologic doses. DHT had little to no activity on PR-A. On PR-B in HeLa cells, MPA had transcriptional activity equivalent to or lower than that of R5020 at all doses. DHT was active at the 1000 nmol/l dose. In T47D-Y cells, MPA had lower transcriptional activity at all doses through both PR-A and PR-B, at least on this synthetic promoter. DHT exhibited no transcriptional activity through PR-A but had some activity through PR-B.

### Expression profiling of acute progesterone versus medroxyprogesterone acetate in progesterone receptor-positive breast cancer cells

**Figure 3:**
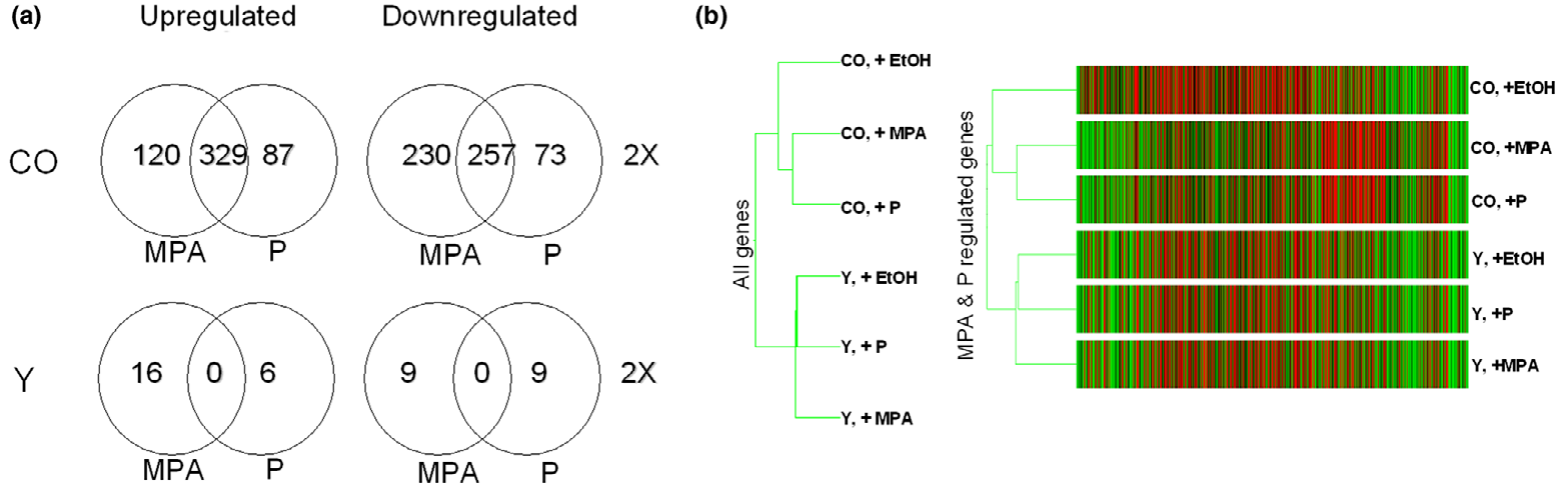
Expression profiles of endogenous PR-regulated genes. Shown are expression profiles of PR-regulated endogenous genes in breast cancer cell lines, with physiological progesterone versus MPA concentrations compared. **(a) **Venn diagrams showing gene number per condition. PR-positive T47Dco cells (CO) and control PR-negative T47D-Y (Y) cells were treated with 10 nmol/l progesterone or MPA for 6 hours in triplicate, time-separated experiments. Microarray analysis using Affymetrix U-133 2 plus gene chips, displaying about 47,000 genes, was performed. Data were analyzed using Gene Spring software and statistical analysis was conducted using a one-way analysis of variance, with *P *< 0.05 considered statistically significant for genes regulated at least 2.0-fold. Detailed tables showing complete gene lists are available as Additional files 1 and 2. **(b) **Dendrograms of PR-regulated genes showing relationships among treatment groups. Gene Spring 6.0 was used to classify all genes 'present' and all MPA- and progesterone-regulated genes. On the left is shown the relatedness among groups for all genes. On the right is a heat map of progestin regulated genes: black = average expression; red = above average; and green = below average. Each column represents a single gene; each row is a cell type and treatment group. DHT, dihydrotestosterone; MPA, medroxyprogesterone acetate; PR, progesterone receptor.

To evaluate in detail the differences, if any, between physiologic concentrations of progesterone and MPA, we used expression profiling (Fig. 3a) at a sufficiently early time point to assess direct transcriptional effects of the hormones [9] while avoiding problems of progesterone metabolism [34]. Wild-type T47Dco breast cancer cells containing equimolar levels of PR-A and PR-B [46] were treated with 10 nmol/l progesterone or MPA for 6 hours. T47D-Y, a PR-negative T47Dco subline, served as the control. RNA from triplicate, time-separated data points was used for analysis of gene chips interrogating about 47,000 human transcripts. Statistically significant 2.0-fold regulation was chosen as the cutoff. Venn diagrams presented in Fig. 3a summarize the results.

Using a 2.0-fold cutoff, in T47Dco cells a total of 1096 (2.3%) transcripts were found to be regulated by one or both hormones. Of these, 49% were upregulated and 51% were downregulated. Among upregulated genes, 329 (61%) were controlled by both progesterone and MPA, with the extent of regulation ranging between about 38-fold and 2.0-fold. Similarly, among downregulated genes 257 (46%) were controlled by both hormones, with the extent of regulation ranging from about 14-fold to 2.0-fold. When the 2.0-fold restriction was slightly relaxed, the extent of overlap between progesterone and MPA was even greater. For example, of the 120 genes regulated 2.0-fold by MPA, 60 genes were also regulated by progesterone if a 1.5-fold cutoff was allowed.

In summary, we found remarkable congruence in genes regulated by progesterone and MPA. Detailed gene lists are available in Additional file 1. These tables demonstrate that both the gene expression profiles and extent of regulation were similar between the hormones. The few genes regulated uniquely by either progesterone or MPA (Fig. 3a) were regulated at much lower levels. Note also that PR-negative T47D-Y cells were included as controls and exhibited very little progestin regulation, as was expected. We speculate that the few genes that appear to be regulated do so via a membrane receptor [51].

The dendrogram presented in Fig. 3b, assembled from the sum of all MPA + progesterone regulated genes demonstrates graphically the remarkable similarity between the two hormones, which segregate PR-positive, hormone-treated cells into a different branch from PR-positive, untreated cells. Also of interest is the fact that hormone treatment has little effect in the absence of PR [51] and that PR positivity, even in the absence of hormone, alters cells sufficiently to segregate them into a different branch of the dendrogram. This unliganded effect of PR has been reported in detail elsewhere [52].

### Synthetic progestins and glucocorticoid receptors

**Figure 4:**
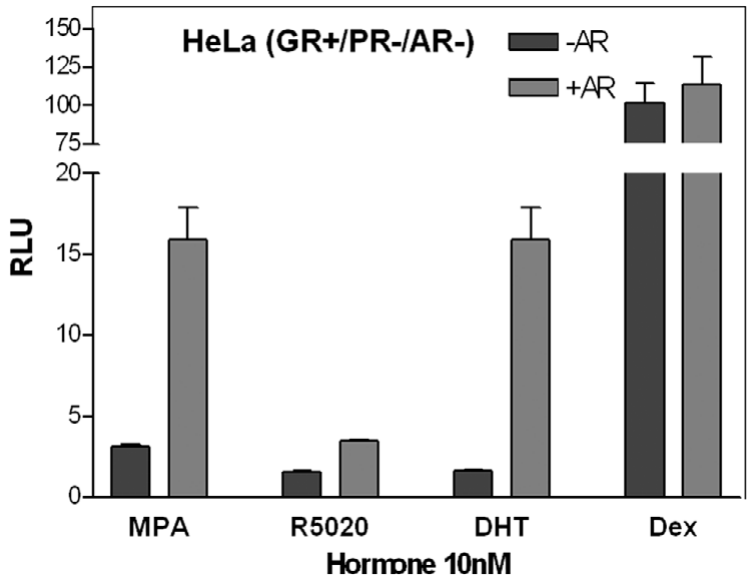
Glucocorticoid and androgenic activity of the synthetic progestins and Dexamethasone. HeLa cells (GR^+^, PR^-^, AR^-^) were transfected with or without an AR expression vector, PRE_2_/luciferase construct and Renilla control, and treated for 20 hours with 10 nmol/l MPA, R5020, DHT, or dexamethasone. Relative luciferase activity was measured. Stippled bars are GR^+ ^cells lacking AR; grey bars are GR^+ ^and AR^+ ^cells. AR, androgen receptor; DEX, dexamethasone; DHT, dihydrotestosterone; GR, glucocorticoid receptor; MPA, medroxyprogesterone acetate; PR, progesterone receptor; RLU, relative luciferase units.

To ensure that the transcriptional activity observed with MPA in T47Dco cells was not occurring through an alternate steroid receptor, we evaluated the activity of the synthetic progestins on GR in the presence or absence of AR (Fig. 4). GR-positive, PR/AR-negative HeLa cells were transfected with or without AR and treated with 10 nmol/l synthetic hormones. No activity was seen with addition of ethanol or a vector alone control (data not shown). Dexamethasone, a GR agonist, exhibited equivalent robust transcriptional activity in the absence or presence of AR, suggesting that its activity is exclusively through endogenous GR. MPA also had weak GR activity (about 15% that of dexamethasone) at the 10 nmol/l dose. However, addition of AR increased the activity of MPA to levels equivalent to those of DHT. No androgenic activity was seen with R5020 or dexamethasone. Similar results would be expected with progesterone, which has about 3% relative binding affinity to AR [53]. T47D-Y cells, the parental cells of the YA, YB, and Y-AR breast cancer cells, were found to contain little GR mRNA based on microarray data and exhibited no transcriptional activity when treated with dexamethasone (data not shown), allowing us to conclude that the activity of MPA in breast cancer cells occurs through PR and AR and not through GR. This study confirms the slight glucocorticoid but strong androgenic activity of MPA.

### Synthetic progestins and androgen receptors

**Figure 5:**
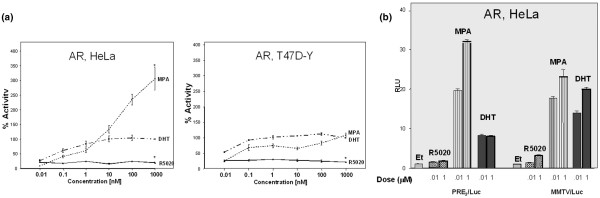
Transcriptional activities of MPA, R5020, and DHT under AR control. **(a) **Cells transiently expressing AR and PRE_2_/luciferase. AR-negative HeLa and T47D-Y cells were transiently transfected with an AR expression vector. Cells were cotransfected with PRE_2_/luciferase construct and Renilla control, treated with varying concentrations of MPA, R5020, or DHT for 20 hours, and luciferase activity was quantified. Data are reported as a percentage of 1 μmol/l DHT (which was set at 100%). *P < 0.05, 1 μmol/l dose of hormone versus 1 μmol/l DHT. **(b) **Androgenic activity on two different promoter constructs. HeLa cells were transiently cotransfected with and AR expression vector and either PRE_2_/luciferase or MMTV/luciferase. Cells were treated with ethanol, or 10 nmol/l and 1 μmol/l R5020, MPA, or DHT for 20 hours, and relative luciferase activity was measured. AR, androgen receptor; DHT, dihydrotestosterone; MPA, medroxyprogesterone acetate; PR, progesterone receptor; RLU, relative luciferase units.

The array data (Fig. 3) showed that MPA and progesterone have similar activity through PR, but clinically the two hormones exhibit different primary and side-effect profiles [32]. We therefore next assessed the activity of MPA on AR (Fig. 5a). HeLa and T47D-Y cells were transfected with an AR expression vector and PRE_2_/luciferase (a consensus binding site for PR, AR, and GR [28-30]) and were treated with increasing concentrations of R5020, MPA, and DHT. Figure 5a shows that, at lower doses, MPA and DHT had approximately equivalent transcriptional activity on AR in both cell lines, and that at pharmacologic doses MPA had exceptionally strong androgenic activity in HeLa cells. R5020 had no androgenic activity in either cell line at all doses.

We then asked whether this androgenic activity of MPA was promoter dependent (Fig. 5b). HeLa cells were transfected with an AR expression vector and two different androgen responsive promoter constructs, namely PRE_2_/luciferase and MMTV/luciferase, and then treated with 10 nmol/l or 1 μmol/l R5020, MPA, or DHT. R5020 had little or no androgenic activity at either dose on either construct. Again, on PRE_2_/luciferase MPA had higher AR activity than DHT, whereas on MMTV/luciferase – a more complex promoter – the AR activity of the two hormones was approximately equal.

### A new, androgen receptor expressing, human breast cancer cell line

**Figure 6:**
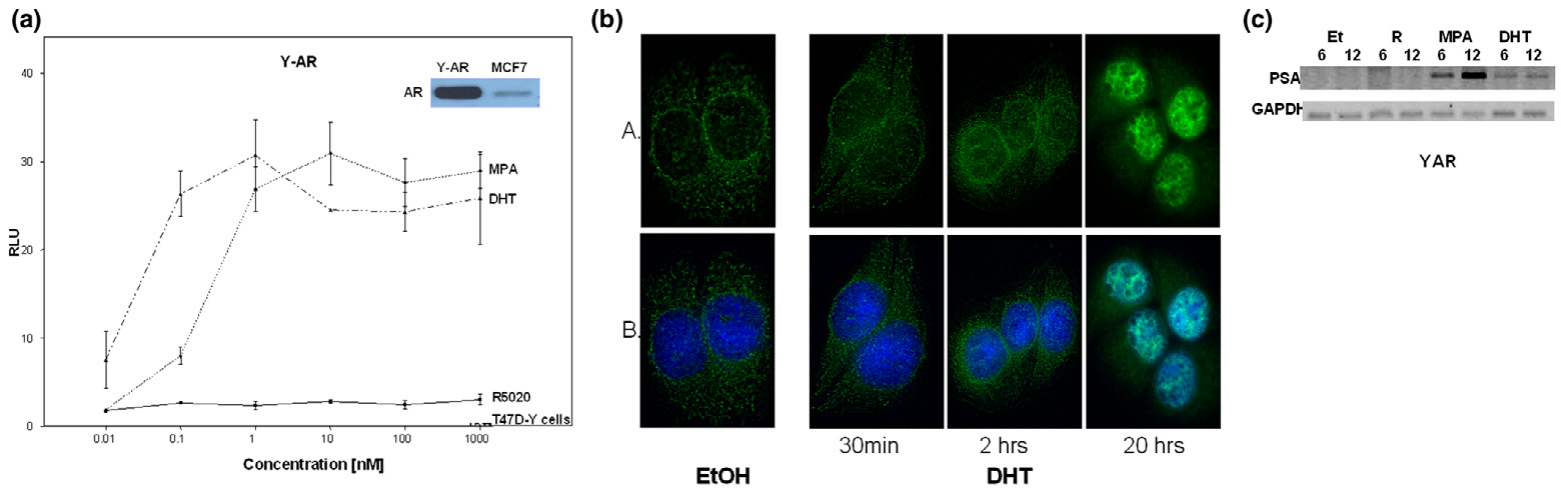
Characterization Y-AR: a new breast cancer cell line stably expressing AR. **(a) **AR levels and function of Y-AR. Inset: whole cell extracts of Y-AR and MCF-7 cells were subjected to SDS-PAGE, and proteins were transferred to nitrocellulose and probed with the anti-AR antibody PG-21 (Upstate Biotechnology). The 110 kDa AR band is shown. Main figure: Y-AR and control T47D-Y cells were transiently transfected PRE_2_/luciferase and treated with DHT, MPA, and R5020 at concentrations from 0.01 to 1000 nmol/l for 20 hours. Luciferase activity is shown as a percentage of 1 mmol/l DHT (which was set at 100%). Data shown are the mean of triplicate determinations ± standard error. **(b) **AR immunocytochemistry in Y-AR cells. Y-AR breast cancer cells were treated with ethanol or 1 μmol/l DHT for 30 min, or 2 and 20 hours. ARs were immunologically visualized using green fluorescent FITC (row A), and the cell nucleus was labeled with blue DAPI (row B). Cells were visualized using confocal microscopy and photographed at 100×, as described in the Materials and method section. **(c) **PCR of the PSA transcript. RNA was harvested from Y-AR cells treated 6 and 12 hrs with ethanol, or 1 μmol/l R5020, MPA, or DHT. RT-PCR was performed using PSA-specific primers. GAPDH is shown as a control. Representative results are shown. AR, androgen receptor; DHT, dihydrotestosterone; EtOH, ethanol; GAPDH, glyceraldehyde-3-phosphate dehydrogenase; MPA, medroxyprogesterone acetate; PR, progesterone receptor; PSA, prostate-specific antigen.

To better study the androgenic properties of synthetic progestins in breast cancer cells, we engineered T47D-Y cells to stably express wild-type AR. The new cells are called Y-AR. Figure 6a (inset) is an immunoblot showing the 110 kDa AR protein in Y-AR cells, equivalent in size to the endogenous AR of MCF-7 cells, which is shown for comparison. The subcellular localization of the stably expressed AR and their response to hormone (Fig. 6B) were analyzed by confocal microscopy. For this, AR were immunologically tagged with green fluorescent FITC and the nuclei labeled with DAPI. A secondary antibody control exhibited no nonspecific binding (not shown). In the absence of DHT, AR were predominantly cytoplasmic. Upon addition of DHT, AR moved to the nucleus within 2 hours and then upregulated by 20 hours. This behavior is similar to that of wild-type AR [48,54]. A western blot was performed that showed that AR levels in Y-AR cells are about the same as in LNCaP cells and similar to PR levels in T47D cells (data not shown).

Functional properties of the stably expressed AR when bound by different ligands were tested in two ways: on the exogenous PRE_2_/luciferase reporter (Fig. 6a) and by regulation of the endogenous prostate-specific antigen transcript (Fig. 6C). Luciferase expression was measured in Y-AR cells treated with increasing concentrations of DHT, MPA, and R5020 (Fig. 6a). Both DHT and MPA generated equivalent maximum transcription levels, with the left shift by DHT suggesting that it has somewhat higher affinity for AR than does MPA. R5020 was completely inactive. No transcription was observed in the parental T47D-Y cells, which lack PR and functional AR.

Prostate-specific antigen is a marker of androgenic activity not only in prostate but also in breast [50,55,56]. Using RT-PCR, we assessed the ability of AR in Y-AR cells to regulate endogenous expression of this gene (Fig. 6c). Cells were treated for 6 or 12 hours with ethanol or hormones. Clearly, R5020 was unable to regulate prostate-specific antigen transcript expression, but both DHT and MPA were able to do so. Interestingly, MPA is reproducibly a strong regulator of this important androgen marker gene.

### Expression profiling of medroxyprogesterone acetate versus dihydrotestosterone in androgen receptor-positive breast cancer cells

**Figure 7:**
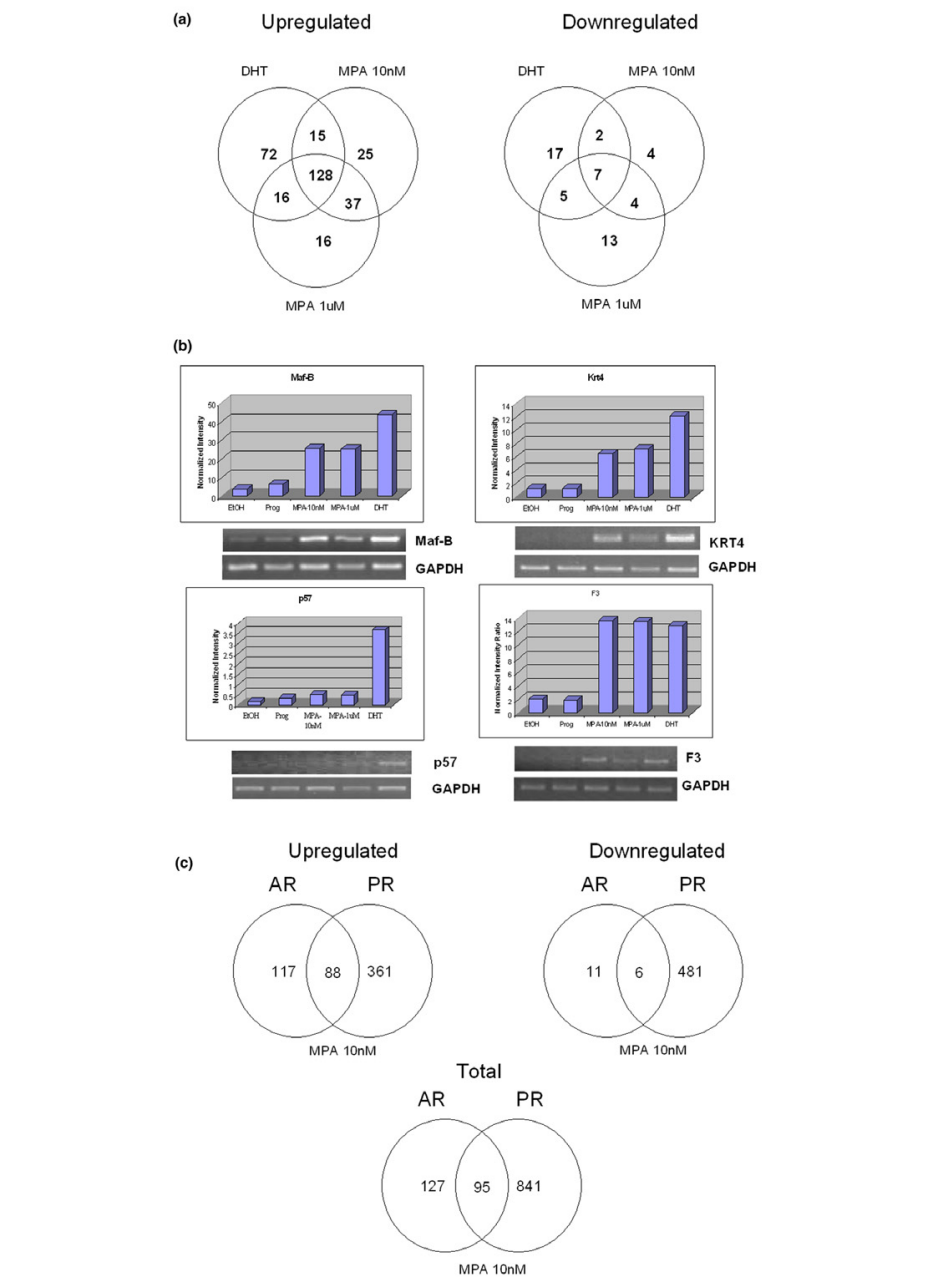
Expression profile of AR regulated genes. Shown are expression profiles of AR-regulated genes, with physiologic DHT versus physiologic and pharmacologic MPA concentrations in Y-AR cells compared. **(a) **Venn diagram comparing the number of genes regulated at least 2.0-fold by low-dose DHT versus low and high dose MPA. Y-AR cells were treated with ethanol, or 10 nmol/l progesterone, MPA, or DHT, or 1 μmol/l MPA for 6 hours in triplicate, time separated experiments. RNA was extracted, derivatized, and used to probe Affymetrix U-133 2 plus gene chips interrogating about 47,000 genes. Data were analyzed using Gene Spring software, and statistical analysis was performed using a one-way analysis of variance, with *P *< 0.05 considered statistically significant for genes regulated at least 2.0-fold. **(b) **Confirmation of the hormonal regulation of four genes identified in panel a. Four genes shown to be hormone regulated in Y-AR cells (i.e. F3, Maf-B, Krt4 and p57) were chosen for further analysis. Bar graphs: the hormonal regulation of the four selected transcripts in Y-AR cells, as assessed by microarray profiling. RT-PCR: Y-AR cells were treated for 6 hours with ethanol, 10 nmol/l progesterone, MPA, or DHT, or 1 μmol/l MPA and RNA was isolated. Primers directed against the transcripts of the four selected genes were used in RT-PCR reactions, and GAPDH was run as an internal control. **(c) **Venn diagrams showing number and overlap of genes regulated by 10 nmol/l MPA in AR^+ ^Y-AR cells versus PR^+ ^T47D cells. Microarray and data analysis were performed as described for Fig. 3a (PR^+^, AR^- ^cells) and panel a above (AR^+^, PR^- ^cells), and data for low-dose MPA in the two cell lines were compared. The 88 genes regulated by MPA through either PR or AR were tabulated (Additional files 1 and 2). The top ranking genes are shown in Table 1. AR, androgen receptor; DHT, dihydrotestosterone; GAPDH, glyceraldehyde-3-phosphate dehydrogenase; MPA, medroxyprogesterone acetate; PR, progesterone receptor.

To understand the androgenic effects of physiologic and high-dose MPA, compared with those of physiologic DHT, Y-AR cells were treated with ethanol, 10 nmol/l progesterone, MPA or DHT, or 1 μmol/l MPA for 6 hours. Gene expression profiles were assessed in triplicate, time-separated experiments, and transcripts regulated at least 2.0-fold in a statistically significant manner through AR were scored (Fig. 7a). Most genes were upregulated. The high dose of MPA was not remarkably different from the low dose, with 165 genes (about 70%) regulated in common, indicating that the 10 nmol/l dose is saturating the AR. Progesterone regulated few genes through AR (not shown) in these PR-negative cells. A comparison of equal doses (10 nmol/l) of MPA and DHT revealed that 143 genes were regulated by both hormones, representing about 60% of all DHT-regulated genes. Thus, MPA is an excellent androgen. Additionally, MPA upregulated a small subset of genes that are not DHT regulated. Similarly, DHT regulated a small subset of genes that are not regulated by MPA. Figure 7a also shows the downregulated genes, which represent less than 17% of total AR regulated genes.

The regulation patterns of four genes were confirmed by RT-PCR (Fig. 7b). Three genes upregulated by both DHT and MPA, namely tissue factor (F3), MAF-B and KRT4, are shown in Fig. 7b. One gene regulated only by DHT – the cyclin dependent kinase inhibitor P57 – is also shown. No regulation of these genes was seen in the parental Y cells, which lack functional AR and PR.

Figure 7c summarizes the progestational versus the androgenic properties of physiological MPA concentrations. Of 566 genes defined as MPA upregulated, about 64% were regulated through PR in T47Dco cells, about 21% through AR in Y-AR cells, and about 15% (88 genes) in both cell lines through both AR and PR. Slightly fewer genes (498) were downregulated by MPA, the striking differences being the much higher percentage of genes downregulated through PR (98%) than through AR (3%). Thus, although MPA upregulated and downregulated many genes via PR, the vast majority of genes regulated by MPA via AR were upregulated only. This is a unique finding and may reflect the ability of MPA-bound AR to recruit coactivators preferentially; this requires independent verification. The upregulated genes were narrowed to 55 genes with regulation that was independently confirmed (i.e. MPA regulated genes that were also regulated by progesterone through PR, and by DHT through AR). Table 2 shows the top 33 of these dual regulated genes, arranged by their fold regulation through MPA compared with the relevant natural hormone in each cell line. Note that for each receptor there is remarkable similarity in the fold regulation observed using MPA compared with the natural hormone. Thus, with regard to the AR-regulated genes, MPA is as good an androgen as is DHT. Also of interest is the fact that, for these dual PR/AR-regulated genes, in some cases the PR but in others the AR were more effective. Explanations for this will require detailed analyses of the promoters in question. The complete table is given in Additional file 2.

## Discussion

### Synthetic progestins, medroxygprogesterone acetate, and progesterone receptor

Synthetic progestins were developed for use in oral contraception and HRT. As a result they were tested for efficacy compared with progesterone, using animal models and physiologic end-points such as inhibition of ovulation and decidualization of the endometrium, focused on the reproductive tract [7]. Consequently, progestins in current clinical use tend to resemble progesterone on the endometrium, with differences, if any, reflecting variations in bioavailability, potency, and metabolism [7]. However, progestins also target organs other than the reproductive tract, including brain, breast, immune, and gastrointestinal systems [57]. This is often reflected in differences in their side-effect profile. For example, compared with progesterone, MPA decreases seizure threshold [58] and incidence of sickle cell crises [59], whereas its androgenic effects may lead to weight gain and lipid profile alterations [60]. Whether these side effects are mediated by PR or other receptors is unclear. The mammary gland was rarely the subject of screening in the initial stages of pharmaceutical progestin development. Nevertheless, it too is a critical target.

The impression that progesterone is an etiologic agent for development of breast cancer is based in part on the increased proliferative activity observed in normal breast during the luteal phase of the menstrual cycle associated with rising progesterone levels [61]. Whether synthetic progestins for HRT can similarly increase breast cancer risk has been evaluated in several studies [62,63], including the recent Women's Health Initiative [3,6]. They show that, in comparison with estrogens alone (including conjugated equine estrogens), synthetic progestins such as MPA increase breast cancer risk [3,6]. The present study used breast cancer models to profile the progestational and androgenic properties of MPA, with the goal of elucidating its gene regulatory properties. We define 'progestational' effects as those that resemble progesterone and are mediated by PR, and 'androgenic' effects as those that resemble DHT and are mediated by AR. Note that this is an arbitrary definition. For example, in transient transcription assays we observed that DHT can signal through PR-B (Fig. 2d,e), an observation that requires further study. If this is indeed the case, then one could argue that this effect of DHT through PR is also 'androgenic'.

The results of our transcriptional assays suggest varying transcriptional effects of hormone in different tissues and with expression of one or both isoforms of PR. The relevance of PR isoform expression in breast cancer has only recently been appreciated [26,64]. Although the present study utilized breast cancer cells that coexpress PR, as is the case in normal breast and in the majority of breast cancers, studies in breast cancer cells expressing only one PR isoform are in progress. We hypothesize that the increase in luciferase activity in cervical cancer compared with breast cancer cells may reflect varying levels of coactivators/corepressors in each tissue, as was demonstrated by Liu and coworkers [65]. In addition, the increased transcriptional activity in wild-type PR-A and PR-B containing cells compared with cells containing only one receptor may reflect the additional contribution of the PR-A/PR-B dimer to transcription.

Here we addressed the global gene regulatory properties of MPA in comparison with those of progesterone in wild-type cells that express equimolar levels of PR-A and PR-B. We found that approximately equal numbers of genes are downregulated and upregulated by each progestin. Because previous studies [8,9] assessing the contributions of individual PR isoforms reported more upregulated genes, the present finding may represent the contribution of the PR heterodimer to gene regulation. We found that almost all transcripts regulated by progesterone were also regulated by MPA, and in most cases that regulatory levels were approximately equal, with some transcript levels altered more extensively by MPA than by progesterone. Although a few genes appeared to be uniquely MPA regulated, their regulation was weak and the significance of this, if any, requires further study. PR-negative T47D-Y cells were tested as a negative control. Very few genes (about 25) were regulated by the progestins in these cells, and we speculate that this may represent actions of alternate steroid receptors such as GR or perhaps membrane-bound PR [51]. Overall, we conclude that with regard to PR, MPA may be a somewhat more potent but otherwise qualitatively similar progestin to progesterone. Its clinical value lies in its resistance to metabolism and more stable pharmacokinetics as compared with progesterone [32]. In studies to be reported elsewhere, we found similarly that several other synthetic progestins in clinical use have expression profiles resembling progesterone [66].

### Androgenicity of medroxyprogesterone acetate

In addition, our studies clearly indicate that, unlike progesterone, MPA is a potent androgen in breast cancer cells. In Y-AR cells treated with 0.1 nmol/l DHT or MPA, we also found that the androgenic activity of both hormones is 80–90% inhibitable by the antiandrogen bicalutamide (10 μmol/l; data not shown). That MPA has androgenic activity has been suspected based on its side-effect profile in other organs [67] at the low concentrations used for oral contraception and HRT. With regard to normal breast, epithelial cells express estrogen receptor (ER), PR and AR, but adjacent myoepithelial cells and stroma express none of the three steroid receptors [68]. In malignancy, grade 1 and 2 ductal carcinoma *in situ *express ER, PR and AR, whereas the majority of grade 3 ductal carcinoma *in situ *are ER and PR negative but continue to be AR-positive [68]. Lea and coworkers [69] quantitated AR by immunohistochemistry in 1026 metastatic tumors and found that AR were present at double the frequency of PR. In fact, in one out of four tumors AR are expressed as the sole sex hormone receptor. The expression of AR in ER-negative tumors is associated with a better disease-free survival [70]. In this regard, it is interesting that addition of testosterone to standard estrogen or estrogen + progesterone therapy reportedly reduces the risk associated with HRT to that of the untreated population [71]. Thus, the expression and activation of AR may play an important role in the development of breast cancers and response to hormone therapies.

The data presented here show that, in human breast cancer cells, physiologic doses of MPA elicit as good an androgenic response as DHT and as good a progestational response as progesterone. We would conclude that at the physiologic doses used for oral contraception and HRT, MPA delivers a powerful androgenic outcome. However, MPA is often given at pharmacologic doses, such as when it is used as a second-line agent for the treatment of metastatic breast cancer and for treatment of early endometrial cancer [39]. Numerous investigators have suggested that the efficacy of MPA at pharmacologic doses in breast cancer treatment is due to activation of AR rather than PR at those doses [37]. Based on our data (Fig. 7a), we would argue that high-dose MPA is no different from low dose MPA. This makes sense if physiologic concentrations suffice to saturate both PR and AR, in which case higher concentrations would not be expected to have further effects. Indeed, our data clearly show that increasing the dose of MPA does not alter its transcriptional profile. Based on this we would argue that there is no rationale for using high-dose MPA for any indication, and suggest that lower doses might suffice for treatment of endometrial and breast cancers. If lower doses are indeed sufficient, then the administration of high doses may only exacerbate side effects without improving the therapeutic index. Further studies evaluating the use of lower doses of MPA in treatment of AR-positive breast cancers would be of interest.

There was some overlap (Fig. 7c) among genes upregulated by MPA *in vivo *through both PR and AR. As we define them, these are 55 genes upregulated by progesterone through PR, and by DHT through AR, with MPA having the ability to activate both sets. We suggest that this blurs the boundaries between androgenic and progestational effects. Further analysis of the promoter sequences and tissue distributions of these 55 genes may suggest a pattern of regulation that allows selective activation by only one receptor *in vivo*. Alternatively, these may be key genes that evolved in both males and females, for regulation by androgens or progesterone, respectively. In summary, more genes are regulated (both up and down) by MPA through PR than through AR. Interestingly, there are genes regulated by DHT that are not regulated by MPA (Fig. 7a). The mechanisms underlying this differential effect also require study. One of these genes is p57, a cyclin-dependent kinase inhibitor with activity that suggests a possible mechanism for inhibition of cell cycle regulation and proliferation by AR in breast cancers.

## Conclusion

The finding that physiologic MPA is both a potent progestin and androgen in the same cell type has important implications for women's health. The strong androgenic effects of MPA when compared with progesterone suggest that physiologic hormone replacement using progestins such as MPA in HRT do not mimic the actions of endogenous progesterone alone. Our data suggest that the findings of the Women's Health Initiative may represent the dual action of MPA on PR and AR. The studies described here are limited to short term treatments *in vitro*, with the limitations thereof. We have begun to investigate the effects of long-term MPA treatment using the new Y-AR breast cancer cells growing as xenografts in nude mice to test further our hypothesis that the activity of MPA in the breast is mediated through its dual function as a progestin and androgen.

## Abbreviations

AR = androgen receptors; DCC = dextran-coated charcoal; DHT = dihydrotestosterone; ER = estrogen receptor; GR = glucocorticoid receptors; HRT = hormone replacement therapy; MPA = medroxyprogesterone acetate; PBS = phosphate-buffered saline; PR = progesterone receptor; PRE = progesterone responsive element; RT-PCR = reverse transcription polymerase chain reaction; RR = relative risk.

## Competing interests

The author(s) declare that they have no competing interests.

## Authors' contributions

RPG designed the studies, created the Y-AR breast cancer cell line, and analyzed the microarray data. BMJ participated in the coordination of the studies, performed the microarray experiments and helped in the analysis of the data. SS performed the tissue culture and assisted in the microarray experiments. KBH conceived the study, participated in its design and coordination, and helped to draft the manuscript.

## Supplementary Material

Additional File 1 Excel files containing tables showing the genes regulated by progesterone and MPA in T47D breast cancer cells: A, MPA upregulated; B, progesterone upregulated; C, MPA downregulated; D, progesterone downregulated; E, upregulated by both progesterone and MPA; F, downregulated by both progesterone and MPA. Click here for file

Additional File 2 Excel files containing tables showing the genes regulated by DHT and MPA in Y-AR breast cancer cells: A, DHT upregulated; B, MPA 1 μmol/l upregulated; C, MPA 10 nmol/l upregulated; D, DHT downregulated; E, 1 MPA μmol/l downregulated; F, MPA 10 nmol/l downregulated; G, upregulated through AR and PR. Click here for file

## Figures and Tables

**Table 1:** RT-PCR primer sets

Gene ID	Forward primer	Reverse primer
KLK3 [72] (GenBank: NM_001468)	TGCGCAAGTTCACCCTCA	CCCTCTCCTTACTTCATCC
Krt4 (GenBank X07695)	GTAGCTACTTCTTGATTTGGGCCTG	ATAATACAGGATCTAGTGGGAGATG
CDKN1C (GenBank: NM_000076)	TGAGAAGTCGTCGGGCGATGTCCCC	GCCGGTTGCTGCTACATGAACGGTC
MAFB (GenBank: NM_005461)	TCAAGTGCGTTCTTTAGACCAATGC	CTGATGCAGGACAAATATCCACAAT
F3 (GenBank: NM_001993)	CACAGAGTGTGACCTCACCGACGAG	GTACTCTTCCGGTTAACTGTTCGG

**Table 2:** Top 33 dual regulated genes

GenBank ID	AR		PR		Description
		
	MPA	DHT	MPA	P	
NM_005627	67.53	51.80	23.33	29.48	Serum/glucocorticoid regulated kinase
AI985987	31.45	33.87	6.39	11.72	Sodium channel, nonvoltage-gated 1, gamma
AI492388	26.17	11.44	37.57	31.08	ti27d10.x1 NCI_CGAP_Kid11 Homo sapiens cDNA clone IMAGE:2131699 3', mRNA sequence.
AV707102	11.91	11.13	4.23	4.04	Pyruvate dehydrogenase kinase, isoenzyme 4
NM_004117	11.10	9.08	3.15	3.21	FK506 binding protein 5
AL556438	10.88	11.23	4.77	3.14	TCDD-inducible poly(ADP-ribose) polymerase
AA404269	8.33	7.36	6.22	5.29	Prickle-like 1 (*Drosophila*)
AB018580	7.54	5.38	4.74	4.69	Aldo-keto reductase family 1, member C3 (3-alpha hydroxysteroid dehydrogenase, type II)
NM_005461	6.82	11.65	15.98	15.80	v-maf musculoaponeurotic fibrosarcoma oncogene homolog B (avian)
NM_001993	6.76	6.39	20.32	12.77	Coagulation factor III (thromboplastin, tissue factor)
NM_001992	6.01	5.99	4.61	2.84	Coagulation factor II (thrombin) receptor
BE219979	5.82	8.07	12.62	12.49	Interleukin 20 receptor, alpha
NM_019018	5.06	4.63	8.18	8.43	Hypothetical protein FLJ11127
NM_006096	4.63	4.34	10.19	10.50	N-myc downstream regulated gene 1
NM_012429	4.42	4.95	4.06	3.76	SEC14-like 2 (S. cerevisiae)
W86302	4.22	3.96	2.58	2.68	FK506 binding protein 5
AF288571	4.07	3.95	2.91	3.38	Lymphoid enhancer-binding factor 1
AW135013	4.00	8.54	16.37	13.28	v-maf musculoaponeurotic fibrosarcoma oncogene homolog B (avian)
BF593382	3.71	3.53	2.16	2.27	Protein kinase, AMP-activated, beta 2 non-catalytic subunit
AF131840	3.61	4.13	3.87	3.14	SPRY domain-containing SOCS box protein SSB-1
AA721025	3.47	3.00	5.59	3.30	*Homo sapiens *transcribed sequence with moderate similarity to protein ref:NP_060265.1 (H.sapiens)
AI753747	3.32	3.23	2.46	2.22	FK506 binding protein 5
N73742	3.31	3.18	3.73	3.45	*Homo sapiens *cDNA FLJ42287 fis, clone TLIVE2005866
AA037766	3.27	2.81	3.17	2.56	*Homo sapiens *LOC374963 (LOC374963), mRNA
AL357503	3.17	4.55	8.33	8.02	Match: proteins: Tr:Q9UJ12 Tr:O43723 Tr:Q9VZN3
NM_004235	3.06	4.89	4.69	4.83	Kruppel-like factor 4 (gut)
BI668074	3.06	4.81	2.52	2.36	603295907F1 NIH_MGC_96 Homo sapiens cDNA
AL562398	2.82	2.24	2.12	2.14	AL562398 Homo sapiens NEUROBLASTOMA COT 25-NORMALIZED
BG150485	2.78	2.88	2.79	2.03	Solute carrier family 6 (neurotransmitter transporter, taurine), member 6
BF056282	2.77	2.43	2.33	2.18	Chromosome 9 open reading frame 52
BF514079	2.73	4.33	8.09	7.66	Kruppel-like factor 4 (gut)

Shown are the top genes and their fold upregulation in Y-AR (AR^+^) and T47D (PR^+^) cells treated with physiologic concentrations of MPA and either DHT or progesterone. AR, androgen receptors; DHT, dihydrotestosterone; MPA, medroxyprogesterone acetate; P, progesterone; PR, progesterone receptor.
